# Exposure to Endocrine Disruptors and Stress Hormones Across Pregnancy Trimesters: Links with Maternal Telomere Length

**DOI:** 10.3390/jox16030082

**Published:** 2026-05-07

**Authors:** Elena Vakonaki, Eleftheria Hatzidaki, Stella Baliou, Maria Marmara, Athanasios Alegakis, Eleftheria Mylonaki, Zoi Volonaki, Fanourios Makrygiannakis, Aristides Tsatsakis, Manolis N. Tzatzarakis

**Affiliations:** 1 Laboratory of Toxicology, School of Medicine, University of Crete, 71003 Heraklion, Greece; 2 Department of Neonatology, Neonatal Intensive Care Unit, University Hospital of Heraklion and School of Medicine, University of Crete, 70013 Heraklion, Greece; 3 Department of Obstetrics and Gynecology, University Hospital of Heraklion, 71500 Heraklion, Greece; 4 Human Development and Health Science Faculty, University of Ecotec, Samborondon EC092302, Ecuador; 5 Biomedical Science and Technology Park, First State Medical University, Moscow 119991, Russia

**Keywords:** hair, telomeres, parabens, triclosan, biomonitoring, cortisol, cortisone, pregnancy

## Abstract

Background: Exposure of pregnant women to stress and endocrine-disrupting chemicals (EDCs) during pregnancy can have a substantial impact on mother and infant health. We investigated the concentrations of EDCs, such as parabens (PBs) and triclosan (TCS), as well as stress hormones (cortisone and cortisol), across pregnancy trimesters and examined their associations with maternal average telomere length (TL). Methods: Hair samples from 49 postpartum women were analyzed using liquid chromatography–mass spectrometry (LC-MS) to quantify EDCs and stress hormone concentrations. Results: The mean methyl paraben concentrations in the hair of postpartum women were prevalent across all pregnancy trimesters, while butyl paraben was detected at the lowest levels. The mean concentration of PBs followed the order methyl > propyl > ethyl > benzyl > butyl paraben across pregnancy trimesters. We found that ethyl paraben and triclosan were each positively and significantly associated with cortisol levels in postpartum women’s hair. Consistent with this, the mean cortisone concentration gradually increased from the first to the third pregnancy trimester, whereas cortisol reached the highest mean concentration at the second trimester. A significant positive association between cortisol and cortisone levels was observed. Further analyses revealed that mothers’ average TL was positively associated with ethylparaben and triclosan levels and inversely associated with benzylparaben levels. Last but not least, we found that cortisol/cortisone levels were positively associated with postpartum women’s TL in a statistically significant manner. Conclusions: In the present study, prenatal exposure to stress hormones and EDCs appears to exert a statistically significant impact on maternal TL dynamics.

## 1. Introduction

During pregnancy, total cortisol levels increase markedly, primarily due to an estrogen-dependent rise in hepatic corticosteroid-binding globulin (CBG), which transports total cortisol in the bloodstream [1]. However, only a small fraction of total cortisol (free cortisol) is active [2]. Free cortisol is a vital component of the hypothalamic–pituitary–adrenal (HPA) axis, playing a crucial role in regulating metabolism, stress, and the immune response [3,4]. During gestation, the fetus is protected from maternal glucocorticoid excess by elevated cortisone levels in the amniotic fluid due to increased placental 11β-hydroxysteroid dehydrogenase 2 (11β-HSD2) activity, which converts cortisol into cortisone [5]. As a result, cortisol and cortisone are considered reliable biomarkers for assessing long-term glucocorticoid exposure [6].

In addition, maternal stress can be further exacerbated by environmental exposures, particularly to endocrine-disrupting chemicals (EDCs) during pregnancy. These EDCs may disrupt glucocorticoid homeostasis and maternal stress responses during pregnancy by interfering with endocrine signaling pathways, such as the HPA axis [7]. EDCs are exogenous substances that alter the normal endocrine system function and disrupt hormonal balance [8]. In particular, EDCs can mimic, block, or alter the metabolism of natural hormones such as estrogens, androgens, and thyroid hormones [9]. Regarding EDC-related complications in pregnant women, gestational diabetes mellitus (GDM) and hypertensive disorders, including preeclampsia and thyroid dysfunction, are common adverse outcomes in EDC-exposed pregnant women [10,11].

Parabens (PBs) constitute a group of EDCs that are known for their antimicrobial preservative properties [12]. They are commonly used in personal care products applied to the skin and in paper packaging materials for consumer food products [8,13]. Methylparaben (MenP) and propylparaben (ProP) are among the most frequently used PBs. Structurally, PBs are esters of p-hydroxybenzoic acid with alkyl chains ranging from methyl to benzyl groups [14]. The widespread use of PBs leads to long-term exposure, especially during critical developmental periods such as pregnancy [15]. In a molecular setting, PBs have been reported to exhibit weak estrogenic activity by binding to estrogen receptors (ERs), and they may also affect the HPA axis, potentially disrupting cortisol synthesis [16,17]. Notably, the estrogen receptor binding affinities of PBs are 10,000–100,000 times lower than that of 17β-estradiol [18,19].

Triclosan (TCS) is another EDC with antimicrobial properties and is widely used in personal care products, detergents, medical devices, and plastic kitchenware due to its antibacterial and antifungal activity [13]. Experimental evidence suggests that TCS inhibits 11β-HSD2 in human placental syncytiotrophoblasts, which may result in increased fetal exposure to cortisol and potential impairment of fetal development [20].

Emerging evidence suggests that exposure to EDCs during the prenatal period plays a crucial role in orchestrating telomere length (TL) dynamics, highlighting the potential of telomeres as biomarkers of environmental exposure [21]. In particular, stress hormones may also influence telomere regulation during pregnancy, as increased maternal oxidative stress, inflammation, and reduced telomerase activity have been associated with accelerated cellular aging [21,22,23,24]. Although a few studies have examined the independent effects of stress hormones or EDC exposure on telomeres, there is an urgent need to understand how the combined exposure of postpartum women to EDCs and stress hormones during pregnancy affects telomere dynamics in this population.

Our hypothesis is that increased EDC exposure may influence the HPA axis during pregnancy, pointing to the role of stress hormones (cortisol and cortisone) acting as mediators in the relationship between EDC exposure and telomere dynamics across pregnancy trimesters. For this reason, the first aim of our study is to evaluate the retrospective cumulative exposure of pregnant women to various EDC and maternal stress hormones (cortisol and cortisone) using liquid chromatography–mass spectrometry (LC-MS) across the three trimesters of pregnancy. In particular, cortisone, cortisol, methylparaben (MenP), ethylparaben (EthP), propylparaben, butylparaben (ButP), benzyloparaben (BenP) and triclosan (TCS) concentrations were measured in postpartum women’s hair. The present study also aims to examine the associations of prenatal maternal stress hormones and selected EDCs on postpartum women’s TL values. As a result, this study offers new insights into how environmental and hormonal factors may influence maternal telomere dynamics during pregnancy.

## 2. Materials and Methods

### 2.1. Sampling

The University Hospital of Heraklion, Crete, served as the recruitment site for the study population. The Department of Neonatology and the Neonatal Intensive Care Unit (NICU) at the University Hospital of Heraklion collected head hair and blood samples from postpartum women from October 2022 to February 2023. The Research Ethics Committee of the University of Crete (112/08.09.2021) granted ethical approval for this study. All the procedures were conducted anonymously, as instructed by the EU General Data Protection Regulation (GDPR; available online: https://gdpr-info.eu/, accessed on 15 April 2026). All the postpartum women (49 participants) consented to provide hair samples for assessment of EDC and cortisol/cortisone concentrations. A subset of patients from a previously published cohort [25] was included in this study to evaluate EDC and stress hormone deposition not reported before.

The total length of head hair samples was collected; however, only the first 9 cm proximal to the scalp were used for analysis, corresponding to the pregnancy period, considering the mean growth rate of head hair (1 cm/month). The hair samples were cut using steel scissors, wrapped in aluminum foil, and stored in an envelope in the dark at room temperature until analysis.

At delivery, blood samples were collected from mothers to determine telomere length (TL). All the samples were transferred to the Laboratory of Toxicology, Medical School, University of Crete, and the blood samples were stored at −20 °C until analysis.

### 2.2. Reagents

Cortisone, cortisol, parabens, triclosan, and ammonium acetate was purchased from Sigma-Aldrich (St. Louis, MO, USA); methanol and acetonitrile (LC-MS grade) from Fischer Chemicals, (Loughborough, UK); phenobarbital (internal standard―IS) from Lipomed AG, (Arlesheim, Switzerland); and ultrapure water, was obtained using a Merck Direct-Q 3UV water purification system (Darmstadt, Germany).

### 2.3. DNA Extraction-qPCR Methodology

The applied protocol and the results of the analysis of the blood samples were previously reported [25]. In brief, the QIAamp DNA Mini kit (Cat. No. 51306, QIAGEN, Hilden, Germany) was used to extract genomic DNA following the manufacturer’s instructions. TL was measured using the Relative Human Telomere Length Quantification qPCR Assay Kit (ScienCell, Cat. No. 8918, Carlsbad, CA, USA) according to the manufacturer’s guidelines [25,26]. As specified in the kit’s protocol, the average TL at each chromosomal end was determined after the qPCR reaction using an Agilent Technologies Stratagene Mx3005P machine (Agilent Technologies, Santa Clara, CA, USA) [25,26]. The kit provided all the required details for the calculation of the average TL across the samples.

### 2.4. Endocrine-Disrupting Chemicals and Stress Hormone Extraction from Hair

The first 9 cm of the total hair length were used for analysis and were divided into 3 cm segments (3 segments) corresponding to each trimester of the gestation period. Any external contamination was removed by washing the hair samples twice with ultrapure water and methanol, and then drying them at 50 °C. The extraction procedure was previously described by Tzatzarakis et al. [27]. In brief, 100 mg of hair was placed in glass screw tubes, and 2 × 2 mL of methanol and 25 ng of IS were added. The extraction was carried out in an ultrasonic water bath for 2 × 2 h with periodic mixing with a vortex system. The organic layers were collected, combined and evaporated to dryness under a stream of nitrogen at 35 °C. Then, 100 ul of methanol was added to the residues, and the solution was transferred into 2 mL vials with inserts for LC–MS analysis.

### 2.5. Liquid Chromatography Mass Spectrometry Analysis

The analysis was performed with an LC-MS system (Shimadzu, Kyoto, Japan, 2010 EV). The separation of the analytes was achieved on a Supelco Discovery column C18 (250 mm, 4.6 mm, 5 μm; Sigma-Aldrich, St. Louis, MO, USA) at 30 °C. The total flow rate was set at 0.6 mL/min. As gradient solvents, 5 mM ammonium acetate (solvent A) and acetonitrile with 0.1% formic acid (solvent B) for parabens and triclosan analysis, and water (solvent A) and methanol with 0.1% formic acid (solvent B) for cortisol and cortisone analysis were used.

For the detection of the analytes, an atmospheric pressure chemical ionization (APCI) and a quadrupole mass filter in negative selected ion monitoring (SIM) mode were used. The *m*/*z* ions for the monitoring of the analytes and the corresponding retention times (min) are presented in Table 1. The interface, curved desolvation line (CDL) and heat block temperatures were set at 400 °C, 200 °C, and 200 °C, respectively; the detector voltage at 1.5 kV; and the nebulizing gas flow at 2.5 L/min. The retention times and the monitoring *m*/*z* ions (quantifier and qualifier ions) of each compound are presented in Table 1.

### 2.6. Statistical Methods

Levels of parabens, triclosan, cortisone and cortisol were expressed using means, standard deviations, and medians with quartiles. Bivariate associations were expressed using Spearman’s rho coefficient. A nonparametric Friedman’s test was applied to assess differences between cortisone and cortisol in hair across trimesters. Analyses were performed in IBM SPSS Statistics 24.0 statistical software, and graphs were made using an EXCEL spreadsheet. A level of 0.05 was set as significant.

## 3. Results

### 3.1. Method Validation

The applied analytical protocols are validated according to the basic analytical parameters of linearity, limits of determination and quantification, recovery and accuracy. The multistandard solutions of the analytes were prepared in methanol. The hair samples were spiked at 0, 5, 10, 25, 50 and 100 pg/mg for cortisol and cortisone, and at 0, 25, 50, 100, 250 and 500 pg/mg for parabens and TCS. The spiked hair was treated using the same extraction protocol as authentic hair. The obtained calibration curves were used both for the evaluation of the method and for the calculation of the analyte’s levels in mothers’ head hair samples. The coefficients r2 of the spiked samples curves were above 0.98 for all the analytes (Table 1), the % recovery values ranged from 79.0% (for cortisol) to 111.1 (for ProP and TCS) and the % accuracy values ranged from 99.7% (for BenP) to 113.1 (for ButP) (Table 1).

### 3.2. Characteristics of the Participants

A total of 49 postpartum women participated in this study and had a singleton pregnancy. The median age of the participating women was 34.0 (IQR: 30.0–37.0), with a mean age of 33.9 ± 6.1 years. An increase in median body mass index (BMI) of 3.7 (2.3–5.2) Kg/m^2^ was observed during pregnancy, while the weight difference in Kg was 9.8 with a range of 6.5 to 14.5 (Table 2). The percentage of women who reported smoking before pregnancy dropped from 17 (34.7%) to 9 (18.4%), indicating a 53.0% reduction in smokers. Characteristics of pregnancy record that 35 (71.4%) of the women had a natural conception and the preterm deliveries were 28 (57.14%). In 26 cases (53.1%), a pregnancy disorder or complication was recorded, and 17 of them (34.7%) were admitted to the hospital (Table 2).

The use of different daily products such as personal hygiene/care products and food packaging materials was recoded as never/rarely and often/always. Responses in the often/always category included cosmetics (98.0%), soaps (75.5%), nail varnish (40.8%) and hair care products (30.6%). Similarly, food packaging materials or dietary habit responses in the “often/always” category included bottled water (81.6%), plastic containers (61.2%), cling film (61.2%), soft drinks consumption (40.8%), snacks consumption (29.2%) and canned foods (28.6%).

### 3.3. Cortisol and Cortisone Monitoring Results

**Figure 1:**
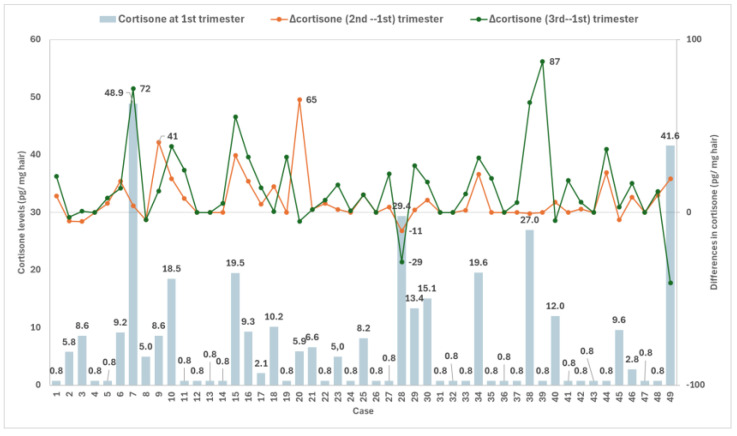
Cortisone levels in hair across pregnancy trimesters. X-axis refers to cases (women); left y-axis shows cortisone levels; right y-axis indicates the difference in cortisone levels relative to the 1st trimester. The vertical light blue bars represent cortisone levels in the 1st trimester, the orange line represents the difference between the 2nd and 1st trimester Δcortisone (2nd–1st) and the green line represents the difference between the 3rd and 1st trimester Δcortisone (3rd–1st).

In Figure 1, levels of cortisone in the first trimester and the differences from the second trimester Δcortisone (2nd–1st) and the third trimester Δcortisone (3rd–1st) are shown. All the measurements were estimated using cortisone imputed values of 0.8 pg/mg hair for non-detected samples. The mean levels of cortisone in the first trimester were 7.4 ± 10.7 pg/mg with a median of 0.8 pg/mg and an interquartile range from 0.8 to 9.3 pg/mg. Levels of cortisone in the second trimester showed a mean of 14.2 ± 18.2 (median: 6.1, interquartile range: 0.8–22.4 pg/mg), showing a mean increase of 6.9 ± 13.1 pg/mg. Similarly, measurements of cortisone during the third trimester showed a mean of 20.9 ± 26.4 (median 11.6, interquartile range: 0.8–23.2 pg/mg), representing an increase of 13.5 ± 22.6 pg/mg. A significant difference in the cortisone levels across different pregnancy trimesters was observed based on the non-parametric Friedman’s test for repeated measurements (χ^2^(2) = 31.59, *p* < 0.001).

**Figure 2:**
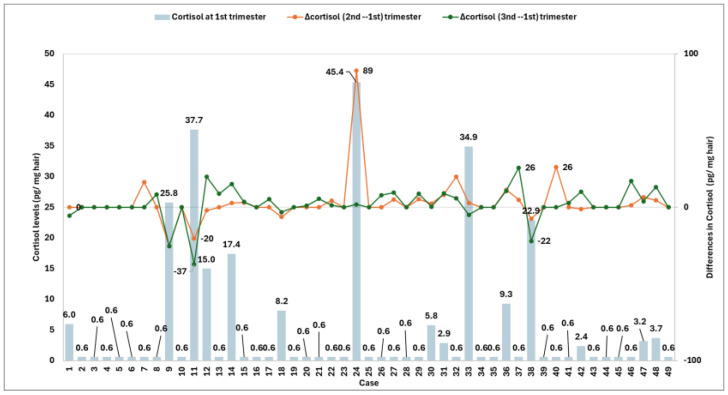
Cortisol levels in hair across pregnancy trimesters. X-axis refers to cases (women); left y-axis shows cortisol levels in the 1st trimester; right y-axis indicates the difference in cortisol levels relative to the 1st trimester. The vertical light blue bars represent cortisol levels in the 1st trimester, the orange line refers to the difference in cortisol levels between the 2nd and 1st trimester Δcortisol (2nd–1st) and the green line represents the difference between the 3rd and 1st trimester Δcortisol (3rd–1st).

**Figure 3:**
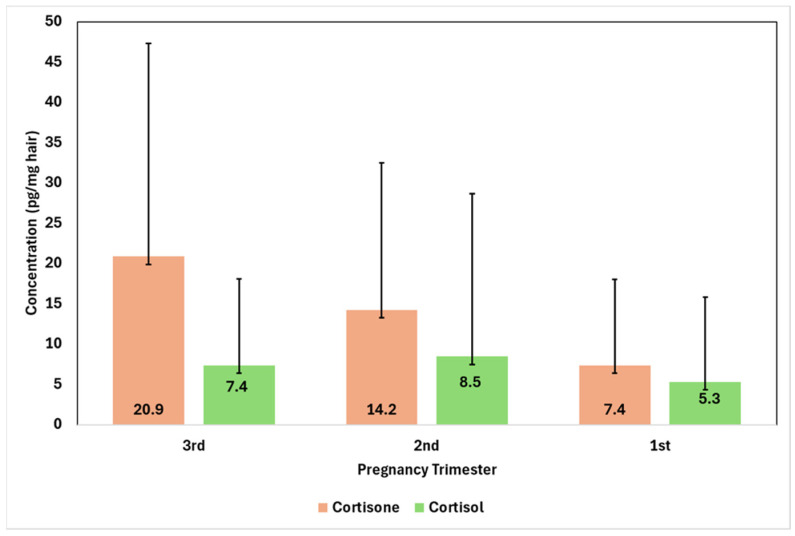
Bar chart of cortisone and cortisol levels in the mother’s hair expressed as mean ± SD of measurements.

Figure 2 shows levels of cortisol in the first trimester of pregnancy and the differences from the second trimester (Δcortisol, 2nd–1st) and the third trimester (Δcortisol, 3rd–1st). All the measurements were estimated using imputed cortisol values of 0.6 pg/mg hair for non-detected samples. Mean levels of cortisol in the first trimester were 5.3 ± 10.5 pg/mg with a median of 0.6 pg/mg and an interquartile range from 0.6 to 3.2 pg/mg. Levels of cortisol in the second trimester showed a mean of 8.5 ± 20.2 (median: 0.6, range: 0.6–8.3 pg/mg hair with a mean increase of 3.1 ± 14.7 pg/mg). Similarly, measurements of cortisol during the third trimester showed a mean of 7.4 ± 10.7 (median 1.7, range: 0.6–9.4 pg/mg) with a mean increase of 2.1 ± 10.2 pg/mg. Differences in cortisol levels across trimesters were significant based on Friedman’s test for repeated measurement χ^2^(2) = 16.91, *p* < 0.001. Figure 3 presents the mean levels and standard deviation of cortisol and cortisone during the pregnancy trimester.

### 3.4. Parabens and Triclosan Monitoring Results

The summary data of EDC head hair concentration levels per trimester of pregnancy are presented in Table 3. There is no significant change in the EDC levels per trimester of pregnancy for MenP (*p* = 0.521), EthP (*p* = 0.805), BenP (*p* = 0.468), ButP (*p* = 0.607) and TCS (*p* = 0.152). Significant differences between trimesters are found for ProP (*p* = 0.049). When a Bonferroni adjustment or Benjamini–Hochberg/false discovery rate (FDR) adjustment was applied, there were no significant differences between EDCs per trimester of pregnancy.

### 3.5. Correlation Between Monitoring Data and Telomere Length

The cortisol and cortisone levels were strongly positively associated (r = 0.73, *p* < 0.001). The cortisol levels were associated with the EthP levels (r = 0.28, *p* = 0.002) and TCS levels (r = 0.22, *p* = 0.011) in the hair of the postpartum women. Additionally, the mothers’ TL values were associated with EthP (r = 0.30, *p* = 0.001) and TCS (r = 0.44, *p* < 0.001). Finally, the maternal TL values were positively associated with their hair cortisone levels (r = 0.24, *p* = 0.002) and cortisol levels (r = 0.29, *p* < 0.001) and negatively associated with BenP (r = −0.26, *p* = 0.008) (Table 4).

In addition, the correlation between the TL values in the postpartum women and age was not statistically significant (r = 0.065, *p* = 0.657), as the pregnant women were homogeneous in age, with 60.0% of the participants aged between 30 and 40 years. In line with this, emerging evidence suggests that higher maternal pre-pregnancy body mass index (BMI) was associated with shorter telomere length in offspring tissues, such as cord blood, placenta, and amniotic fluid [28,29,30]. In our study, the multiple regression analysis was applied, taking into consideration BMI before pregnancy. On one hand, the TL values of the postpartum women were positively associated with TCS (b = 1.6, 95%CI: 1.1–2.2, *p* < 0.001), cortisol levels (b = 23.4 (95%CI: 11.6–35.2, *p* < 0.001) and EthP levels (b = 0.4, 95%CI: 0.1–0.8, *p* = 0.025), adjusted for maternal pre-pregnancy BMI. On the other hand, the TL values of the postpartum women were negatively associated with the BenP levels (b = −3.6, 95%CI: −6.0 to −1.2, *p* = 0.004) and BMI before pregnancy (b = −46.7, 95%CI: −87.7 to −5.6, *p* = 0.026) (Table 5).

## 4. Discussion

### 4.1. Hair Biomonitoring of Endocrine-Disrupting Chemicals and Stress Hormones as Well as Their Association

Although published hair biomonitoring data are limited in pregnant populations [13,31,32], our data provide significant insights into the levels of maternal stress hormones and EDCs in the postpartum women’s hair across the three trimesters of pregnancy.

Hair serves as a reliable matrix for the retrospective assessment of cumulative EDC exposure during pregnancy, due to its non-invasive nature, stability and ability to track long-term exposure rather than short-term fluctuations [8]. In this regard, the quantification of EDCs and glucocorticoids in hair contributes to assessing their cumulative burden, avoiding the need to repeat sampling. Interestingly, the quantification of EDCs, cortisol and cortisone in a pregnancy-trimester-specific manner can be represented, only using hair biomonitoring. In addition, hair analysis enables the retrospective evaluation of chronic physiological stress by measuring glucocorticoids, such as free cortisol (active form) and cortisone, providing insight into long-term stress responses [33,34,35] and eliminating the confounding effects of CBG [34,36,37]. Last but not least, the identification of EDCs and stress hormones in the postpartum women’s hair circumvents the limitations of circadian rhythm and hydration status typically encountered in urine and saliva. When urine and saliva are used for biomonitoring, they capture short-term fluctuations that are related to the hydration status of substances [38]. For example, cortisol and cortisone measured in saliva exhibit pronounced diurnal variation [39].

Initially, we show a significant increase in cortisol concentrations across pregnancy trimesters. In particular, an increase in cortisol levels was observed from the first to the second trimester of pregnancy. In our study, the mean hair cortisol concentration in the postpartum women was proven to range from 5.3 pg/mg in the first trimester to 8.5 pg/mg in the second trimester. The progressive increase in hair cortisol values reported was consistent with hormonal changes that normally occur during pregnancy [40,41]. As pregnancy progresses, the placenta secretes corticotropin-releasing hormone (CRH), which in turn triggers adrenocorticotropic hormone (ACTH) secretion and further activates the maternal HPA axis, leading to a progressive rise in cortisol secretion [40,41]. However, the slight decrease in mean hair cortisol concentration during the third pregnancy trimester could be explained by reduced exposure of the pregnant women to EDCs, increased placental 11β-HSD2 activity, and decreased free cortisol binding to CBG [40,41].

Comparing cortisol levels in previous studies with the results of our study, important similarities and discrepancies are seen. A recent systematic review highlights that the hair cortisol concentrations across three pregnancy trimesters have been shown to align with the mean cortisol values reported for pregnant women, which fall within the concentration range in the first and second pregnancy trimester [42]. In that meta-analysis, the mean hair cortisol concentrations ranged from 0 to 34.15 pg/mg between the first and second trimesters of pregnancy and from 8.59 to 44 pg/mg in the third trimester [42]. Interestingly, this meta-analysis highlighted that the prenatal hair cortisol concentration ranges were greater across trimesters than within trimesters [42]. However, our mean hair cortisol concentrations are slightly higher than those reported by Gelaye’s team, which ranged from 3.38 to 5.59 pg/mg, in pregnant Peruvian women, as measured using liquid chromatography tandem mass spectrometry (LC-MS) [41]. In addition, the mean hair cortisol concentration in the pregnant women in the third trimester was higher than that reported in that study [41]. Interestingly, Dobernecker et al. reported that mean hair cortisol concentration significantly differed among pregnant women of Greece, Spain and Peru, likely reflecting differences in environmental and stress exposure and psychosocial factors [43]. In particular, the Peruvian pregnant women showed the highest mean cortisol concentration in the third trimester, which was associated with cumulative trauma exposure and potential dysregulation of the HPA axis [43].

Secondly, we have quantified cortisone levels in the hair of the women as an additional biomarker of prenatal stress. In stress conditions during pregnancy, the 11β-HSD2 enzyme, which converts cortisol into cortisone, the inactive metabolite of cortisol, seems to be downregulated [44,45]. To support this, our findings have supported a statistically significant increase in cortisone across pregnancy trimesters. In particular, the mean cortisone concentration increased, peaking at 20.9 pg/mg during the third trimester of pregnancy (median 11.6 pg/mg), from 7.4 pg/mg in the first trimester (median 0.8 pg/mg). The gradual rise in circulating cortisone can be attributed to increased estrogen-stimulated CBG generation, increased adrenal activity, and the increased placental CRH generation during pregnancy [46]. In addition, the elevated cortisone levels can be explained by the upregulation of placental 11β-HSD2, which converts cortisol to cortisone, thereby protecting the fetus from excessive active glucocorticoid (cortisol) [5]. As a result, the elevated hair cortisol levels during pregnancy highlight increased placental glucocorticoid turnover.

Thirdly, this study has emphasized the use of hair analysis to monitor long-term EDC exposure across pregnancy trimesters, as assessed by quantifying MenP, EthP, ProP, BenP, ButP, and TCS. In particular, our study highlights the fluctuations in EDC concentrations measured in the hair of the participating women across three pregnancy trimesters, given that PBs are lipophilic and can enter the hair through respiration and blood circulation [47]. The increased EDC detection rates among the participating women across three pregnancy trimesters are consistent with previous research findings supporting the increased exposure to EDCs [48,49,50], with adverse effects on immune, metabolic, and cardiovascular health [51]. In our study, the range of mean MenP concentrations in the hair of the women spanned 4469 to 3240 pg/mg across the first to third trimesters of pregnancy. In the case of EthP, the mean concentration range was 277 to 227 pg/mg in the hair of the postpartum women from the first to the third trimester of pregnancy. Likewise, the mean ProP concentration was detected from 1420 to 2143 pg/mg, the mean BenP concentration was observed from 80.1 to 83 pg/mg, the mean ButP concentration was from 72 to 142 pg/mg and lastly the mean TCS concentration was from 208 to 275 pg/mg in the hair of the postpartum women from the first to the third trimester of pregnancy. The pattern of PB deposition in the postpartum women’s hair was consistent with previous work that proved the order of detected EDCs was MenP > ProP > EthP > BenP > ButP [52]. Additionally, the higher hair PB levels were associated with frequent use of personal care products (such as makeup, hair spray, and sunscreen) [52]. Based on the aforementioned information supporting trimester-specific EDC biomonitoring, a recent study has further elucidated the exposure to PBs, TCS, DDT metabolites and BPS in paired mother–infant dyads, highlighting the significance of maternal EDC exposure during early life and infancy [31].

Furthermore, we sought to evaluate the association of EDCs with stress hormones in the hair of postpartum women, providing insights into these relationships across three pregnancy trimesters. In particular, our results highlight positive, significant associations between EthP and TCS concentrations, and between cortisol concentrations measured in the hair of the postpartum women and the prenatal EthP levels (r = 0.28, *p* = 0.002). Similarly, there was a positive correlation between TCS and cortisol (r = 0.22, *p* = 0.011), but it was weaker than that for EthP in the hair of the postpartum women. Apart from cortisol, our findings have supported that cortisone did not exhibit statistically significant correlations with the majority of EDCs. The significant associations of the selected EDCs with cortisol and not with cortisone in the hair of the postpartum women suggest that the selected EDCs affect the glucocorticoid pathway rather than total glucocorticoid synthesis [53,54], since cortisol is the active form of glucocorticoid hormone and cortisone is considered its inactive metabolite and reversible precursor [55].

Consistent with our findings, a previous biomonitoring study of multiple urinary PBs in Korean pregnant women, using a high-throughput online SPE-LC-MS/MS approach, revealed significant associations of MenP and EthP with stress-related markers, including free cortisol, 8-hydroxydeoxyguanosine (8-OHdG) and malondialdehyde (MDA), suggesting that prenatal exposure to EDCs can have adverse effects on fetal health through the induction of stress responses during pregnancy [56]. In line with this, a recent study showed that prenatal exposure to urinary BPS and some PBs (ProP and MenP) was associated with altered corticosteroid hormone concentrations in hair of pregnant women involved in two European pregnancy cohorts [57]. In particular, increased urinary BPS was associated with higher hair cortisol and 11-dehydrocorticosterone concentrations in pregnant women, whereas increased urinary ProP was associated with lower hair levels of cortisol, cortisone, and 11-dehydrocorticosterone. Additionally, elevated urinary MenP levels were associated with reduced hair cortisol levels in pregnant women [57]. As a result, prenatal exposure to several synthetic phenols was linked to changes in maternal steroid hormone profiles, suggesting that EDCs may interfere with steroid hormone homeostasis during pregnancy [57]. In addition, increased urinary TCS levels were associated with elevated cortisol/cortisone levels [57]. In the molecular setting, prenatal EDC exposure has been reported to inhibit stress hormones by interfering with the glucocorticoid receptor binding, disrupting receptor activation [58], or inhibiting receptor transactivation, thereby modifying the expression of genes involved in signaling pathways [59].

To sum up, the current study highlights important connections between glucocorticoid hormones (cortisol and cortisone) and EDCs, indicating that both physiological stress mechanisms and environmental chemical exposures are connected during pregnancy.

### 4.2. The Association of Endocrine-Disrupting Chemicals and Maternal Stress Hormones with Telomere Length Dynamics in Mothers

A growing body of evidence shows that EDCs can alter glucocorticoid synthesis, secretion, and signaling through their regulation of the HPA axis and on adrenal gland architecture [7,60,61]. Glucocorticoids and telomere dynamics have a complex and context-dependent relationship [62].

Telomeres can serve as important hallmarks evaluating EDC exposure [63]. Pregnancy is a critical susceptible period in which pregnant women and offspring are more prone to EDCs, supporting the concept of aging programming [64]. For example, the prenatal exposure to EDCs has been proven to be associated with changes in TL values in both mothers and their offspring [64]. Interestingly, a significant relationship has been reported between urinary levels of the selected EDCs and TCS during the first trimester of pregnancy and TL dynamics in mother-newborn pairs in a sex-dependent manner [64]. Focusing on the association between stress hormones and telomeres, a meta-analysis has shown that salivary cortisol levels were inversely related to TL values, suggesting that acute stress is associated with telomere shortening [65]. However, measurements of cortisol in urine or blood were not associated with alterations in telomere dynamics [65]. In line with this, previous findings have shown that maternal stress levels significantly affect both maternal and neonatal TL values [66,67], given the positive association between maternal and neonatal TL values reported [25].

To the best of our knowledge, we demonstrate that changes in glucocorticoid homeostasis and TL dynamics in postpartum women are associated with prenatal exposure to specific EDCs. Our study provides evidence of associations between EthP and TCS concentrations in postpartum women’s hair and their cortisol levels and their TL values measured in their blood. In our study, this positive relationship between EthP and TCS and cortisol levels and TL values in hair of postpartum women can be explained by EDC-induced pregnancy-specific endocrine changes rather than from the negative consequences of long-term stress [62]. During pregnancy, the hypothalamic–pituitary–adrenal (HPA) axis is significantly altered, leading to increased cortisol production, which is critical for fetal growth [68]. The potential contribution of EDCs to telomere dynamics in postpartum women can also be accomplished through the following molecular mechanisms. In particular, EDCs can contribute to telomere maintenance in postpartum women because EDCs exert their action by binding to estrogen receptors, as the promoter region of the catalytic subunit of telomerase contains an estrogen response element (ERE) [69]. In such moderate stress conditions, increased glucocorticoid levels do not exert a negative impact on telomere maintenance, as they are linked to compensatory processes during pregnancy, due to transient increases in telomerase activity [70,71]. In addition, cortisol can affect the expression of the catalytic subunit of telomerase, hTERT (human telomerase reverse transcriptase), through the induction of signaling pathways. Under high-cortisol conditions during pregnancy, sustained glucocorticoid signaling has been shown to activate the phosphoinositide 3-kinase (PI3K)-Akt and mitogen-activated protein kinase/extracellular signal-regulated kinase (MAPK/ERK) pathways, which, in turn, are known to upregulate telomerase [72,73]. During pregnancy, elevated estrogen levels also promote adaptive modulation of telomere dynamics through increased telomerase activity as a defense mechanism against potential DNA damage occults [70]. These findings of the potential modulatory effect of EDCs and stress hormones on telomeres may be consistent with the concept of hormesis [74]. On the other hand, prolonged exposure to maternal stress is associated with telomere shortening, oxidative stress, inflammation, mitochondrial dysfunction, telomerase dysregulation, and glucocorticoid signaling, comprising the molecular mechanisms that underlie the effects of long-term stress on telomere dynamics [71,75].

In addition, our findings show that maternal cortisone levels are significantly associated with TL values of postpartum women. From a molecular perspective, telomerase activation may explain the positive association between cortisone and telomere maintenance during pregnancy [76]. In addition, the positive, significant association of cortisone with telomere dynamics in postpartum women can be attributed to its anti-inflammatory and antioxidant actions, which, in turn, may hinder telomere shortening [77,78].

**Figure 4:**
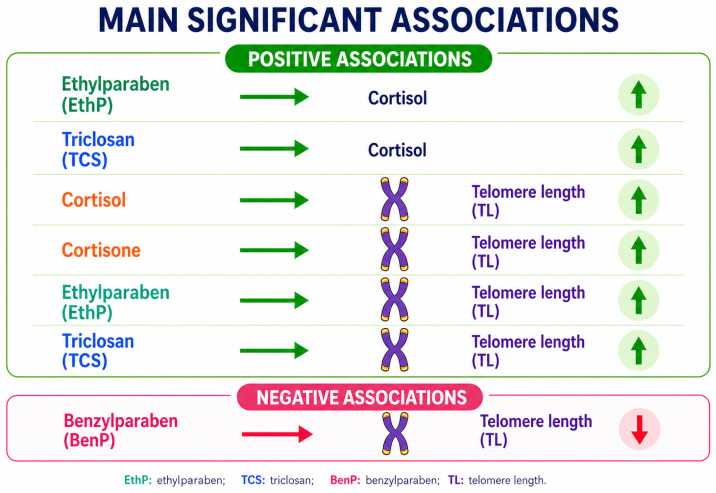
Summary of the main significant associations between EDCs, stress hormones, and maternal telomere length in postpartum women.

To sum up, this study elucidates the impact of prenatal EDC exposure and maternal stress hormones on telomere dynamics in postpartum women, providing deep insights into early-life determinants of biological aging (Figure 4). In more detail, EDC exposure is positively associated with stress hormones (cortisol and cortisone) throughout pregnancy, probably through regulation of the HPA axis. During pregnancy, moderate stress responses elicit compensatory processes, including telomerase activation, thereby contributing to stable TL values in postpartum women and to sustained chromosomal stability in pregnancy conditions.

## 5. Conclusions

Although urine biomonitoring is frequently used to measure EDC levels, this work highlights the need for hair analysis as a biomonitoring tool to assess pregnant women’s cumulative exposure to EDCs and stress hormones across pregnancy trimesters, with the ultimate aim of elucidating the impact of exposure on telomere dynamics in mothers. The current study provides proof of concept regarding EDC patterns across pregnancy trimesters, without any implication regarding EDC health impacts. Initially, hair biomonitoring studies provide information on the EDC burden and stress hormones (cortisol and cortisone) of pregnant women. Secondly, our findings highlight a significant positive association between EthP and TCS levels and cortisol in the hair of postpartum women. Thirdly, our findings support the impact of prenatal EDC exposure and maternal stress on telomere regulation in mothers. Specifically, maternal TCS and EthP are positively associated with maternal TL, whereas BenP is inversely associated with maternal TL.

To summarize, our study’s findings lay the basis for a deeper comprehension of how specific EDC exposures may influence maternal HPA-axis function during pregnancy. In order to gradually mitigate the impact of prenatal anxiety on unfavorable health consequences for the following generation, this knowledge is essential. In addition, our findings provide convincing evidence that prenatal exposure to maternal stress hormones and certain EDCs plays a crucial role in the programming of telomere dynamics in mothers.

## 6. Limitations

This study provides convincing evidence of links among maternal stress biomarkers, EDC and telomere biology across pregnancy trimesters. However, several limitations related to sample size and confounder factors could be considered. Firstly, the study’s small sample size poses a significant challenge, limiting the generalizability of our results. In the future, the larger, prospective longitudinal studies are warranted to validate our findings. Secondly, our study included postpartum women from Greece; our findings might not apply to other populations with various socioeconomic backgrounds due to the unique socioeconomic and cultural context of this community. Thirdly, pregnant women are exposed to multiple EDCs simultaneously [79,80,81,82] and the toxicity of EDCs seems to be affected by their interactions [83]. Fourth, the hair can be used as a biomonitoring tool for EDCs; external environmental pollution may affect hair exposure [32], although strict washing procedures can be used to reduce this effect. Finally, the TL dynamics can be precisely measured in a cell-specific manner using high-resolution quantitative fluorescence in situ hybridization (qFISH), given the limitations of qPCR [84]. In particular, the qFISH approach can provide a precise overall analysis of telomere dynamics, yielding telomere values that are exceptionally short or extremely long at the chromosomal level [84]. Despite these limitations, this study provides strong evidence of dynamic early-life exposure patterns on mothers’ telomere dynamics.

## Figures and Tables

**Table 1:** Retention times (min) and the quantifier and qualifier *m*/*z* ions used for biomonitoring.

	Rt (min)	Quantifier *m*/*z* Ion	Qualifier *m*/*z* Ion	r2 Spiked	LOD (pg/mg)	LOQ (pg/mg)	% Recovery	% Accuracy
Cortisone	9.7	405.2	406.2	0.9999	0.5	1.6	109.8	109.1
Cortisol	10.1	407.2	408.2	0.9977	0.3	1.2	79.0	111.8
MenP	14.1	151.1		0.9902	0.7	2.2	98.9	107.9
EthP	15.9	165.1		0.9864	0.3	1.1	94.5	101.6
ProP	17.6	179.0		0.996	0.1	0.3	111.1	105.7
BenP	19.05	227.1		0.9967	2.9	9.7	79.6	99.7
ButP	19.1	193.1		0.9824	0.8	2.6	96.4	113.1
TCS	23.2	287.0	333.0	0.9976	1.2	4.1	111.1	110.7

LOD: limit of determination; LOQ: limit of quantification.

**Table 2:** Demographic and pregnancy characteristics of women.

		Mean ± SD	Median (IQR)
Demographic	Age (years)	33.9 ± 6.1	34.0 (30–37)
& somatomertics	Height (cm)	166.0 ± 5.5	165.5 (163–170)
	Weight difference (Kg)	10.3 ± 7.3	9.8 (6.5–14.5)
	BMI difference (Kg/m^2^)	3.8 ± 2.7	3.7 (2.3–5.2)
		n	%
Smoking before/after	Yes	17/9	34.7/18.4
Conception	Natural	35	71.4%
	IVF	13	26.5%
Gestation Week	<=36	28	57.1%
	37+	19	38.8%
Pregnancy disorders	Yes	26	53.1%
Admission at hospital	Yes	17	34.7%

BMI: body mass index; IVF: in vitro fertilization.

**Table 3:** Levels of EDCs in postpartum women’s hair per trimester (pg/mg hair).

EDCs	Trimester	% Positive	Mean ± SD	Median (IQR)	*p* *
**MenP**	1st	95.9	4469 ± 11613	199 (52–1487)	0.521
	2nd	93.9	2868 ± 7083	288 (84–1514)	
	3rd	91.8	3240 ± 7469	272 (69–1848)	
**EthP**	1st	83.7	277 ± 685	85 (37–174)	0.805
	2nd	81.6	723 ± 3182	98 (39–208)	
	3rd	83.7.	227 ± 332	95 (43–206)	
**ProP**	1st	87.8	1420 ± 5622	76 (35–177)	0.049
	2nd	83.7	2050 ± 8891	113 (49–309)	
	3rd	87.8	2143 ± 7564	81 (32–357)	
**BenP**	1st	71.4	80 ± 90	45 (31–96)	0.468
	2nd	67.3	86 ± 115	44 (29–92)	
	3rd	63.3	83 ± 89	50 (28–105)	
**ButP**	1st	14.3	72 ± 103	9 (5–194)	0.607
	2nd	10.2	41 ± 44	37 (3–56)	
	3rd	6.1	142 ± 156	87 (21–318)	
**TCS**	1st	93.9	208 ± 241	71 (34–356)	0.152
	2nd	93.9	310 ± 412	116 (51–540)	
	3rd	93.9	275 ± 345	113 (38–447)	

EDCs: endocrine-disrupting chemicals; SD: standard deviation; IQR: interquartile range; MenP: methylparaben; EthP: ethylparaben; ProP: propylparaben; BenP: benzyloparaben; ButP: butylparaben; TCS: triclosan; *: *p*-values from non-parametric Friedman’s test.

**Table 4:** Spearman’s correlation coefficients of cortisone and cortisol levels, maternal median telomere length (TL) vs. endocrine-disrupting chemical (EDC) levels.

	Cortisone		Cortisol		Mother’s TL	
	r	*p*	r	*p*	r	*p*
Cortisone	1.00		0.73	<0.001	0.24	0.002
Cortisol	0.73	<0.001	1.00		0.29	<0.001
MenP	−0.07	0.430	−0.01	0.901	−0.11	0.208
EthP	0.09	0.325	0.28	0.002	0.30	0.001
ProP	−0.10	0.286	0.01	0.915	−0.09	0.306
BenP	0.11	0.278	0.07	0.467	−0.26	0.008
ButP	0.43	0.107	0.17	0.555	−0.04	0.878
TCS	0.12	0.154	0.22	0.011	0.44	<0.001

EDCs: endocrine-disrupting chemicals; TL: telomere length; MenP: methylparaben; EthP: ethylparaben; ProP: propylparaben; BenP: benzyloparaben; ButP: butylparaben; TCS: triclosan.

**Table 5:** Regression coefficients (b) and 95% confidence intervals (CIs) for the association between telomere length (TL) values of postpartum women and their endocrine-disrupting chemical (EDC) exposure, adjusted for maternal pre-pregnancy BMI.

	b	95% LL	95% UL	*p*
Cortisol	23.4	11.6	35.2	<0.001
TCS	1.6	1.1	2.2	<0.001
BenP	−3.6	−6.0	−1.2	0.004
EthP	0.4	0.1	0.8	0.025
BMI before pregnancy	−46.7	−87.7	−5.6	0.026

EDCs, endocrine-disrupting chemicals; LL, lower limit; UL, upper limit; EthP, ethylparaben; BenP, benzyloparaben; TCS: triclosan; BMI, body mass index.

## Data Availability

The data presented in this study are available upon request from the corresponding author due to privacy restrictions.
